# Ten quick tips for biomarker discovery and validation analyses using machine learning

**DOI:** 10.1371/journal.pcbi.1010357

**Published:** 2022-08-11

**Authors:** Ramon Diaz-Uriarte, Elisa Gómez de Lope, Rosalba Giugno, Holger Fröhlich, Petr V. Nazarov, Isabel A. Nepomuceno-Chamorro, Armin Rauschenberger, Enrico Glaab

**Affiliations:** 1 Department of Biochemistry, School of Medicine, Universidad Autónoma de Madrid, Instituto de Investigaciones Biomédicas ‘Alberto Sols’ (UAM-CSIC), Madrid, Spain; 2 Luxembourg Centre for Systems Biomedicine (LCSB), University of Luxembourg, Luxembourg; 3 Department of Computer Science, University of Verona, Verona, Italy; 4 Department of Bioinformatics, Fraunhofer Institute for Algorithms and Scientific Computing (SCAI), Sankt Augustin, Germany; 5 Bonn-Aachen International Centre for IT (b-it), Rheinische Friedrich-Wilhelms-Universität Bonn, Bonn, Germany; 6 Department of Cancer Research, Luxembourg Institute of Health, Strassen, Luxembourg; 7 Dpto. de Lenguajes y Sistemas Informáticos, University of Seville, Seville, Spain; McGill University, CANADA

This is a *PLOS Computational Biology* Methods paper.

## Introduction

High-throughput experimental methods for biosample profiling and growing collections of clinical and health record data provide ample opportunities for biomarker discovery and medical decision support. However, many of the new data types, including single-cell omics and high-resolution cellular imaging data, also pose particular challenges for data analysis. A high dimensionality of the data in relation to small numbers of available samples (often referred to as the p >> n problem), influences of additive and multiplicative noise, large numbers of uninformative or redundant data features, outliers, confounding factors and imbalanced sample group numbers are all common characteristics of current biomedical data collections. While first successes have been achieved in developing clinical decision support tools using multifactorial omics data, e.g., resulting in FDA-approved omics-based biomarker signatures for common cancer indications [1], there is still an unmet need and great potential for earlier, more accurate and robust diagnostic and prognostic tools for many complex diseases.

**Fig 1:**
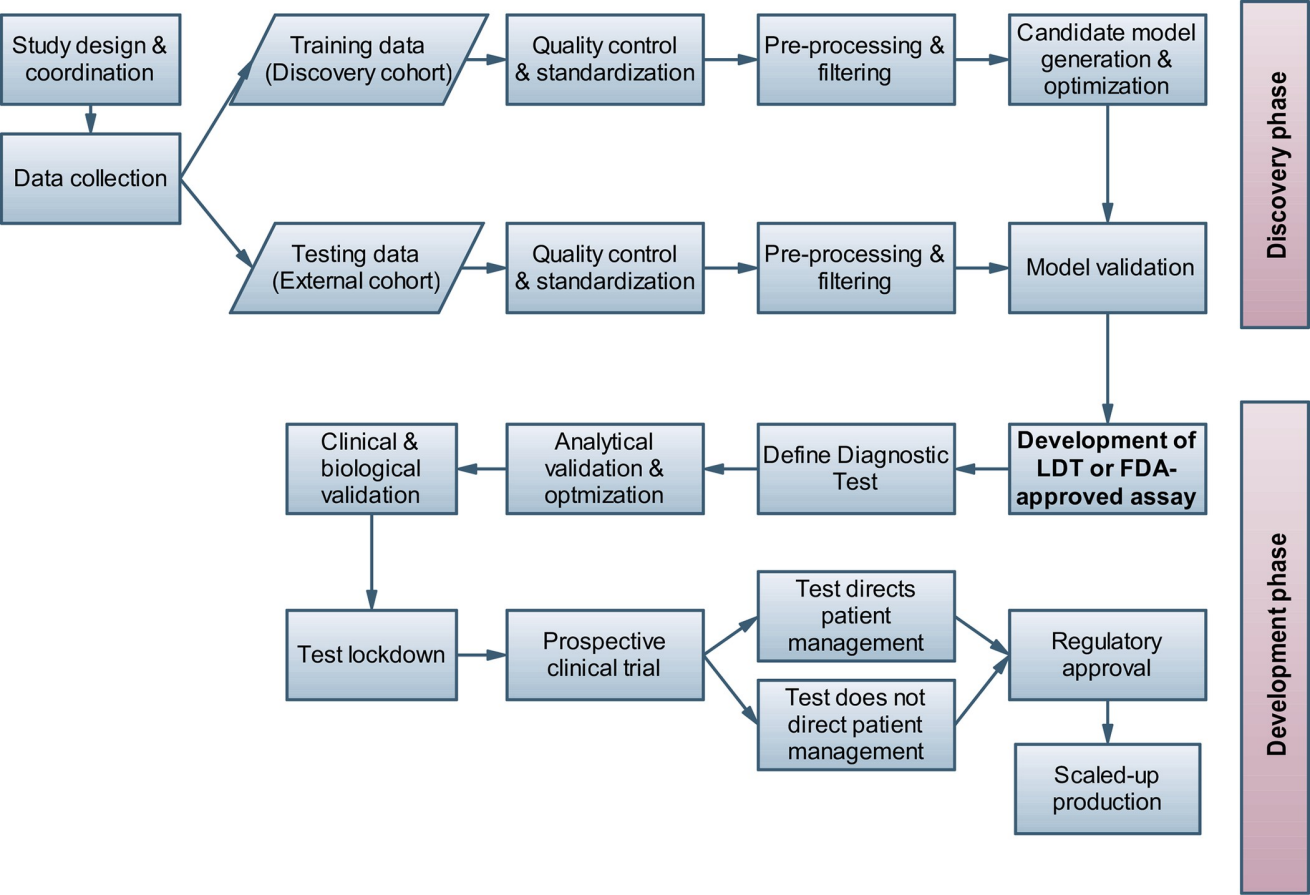
Schematic overview of key steps in a common biomarker test development workflow for patient stratification or disease outcome prediction.

Here, we provide a set of broadly applicable tips to address some of the most common pitfalls and limitations for biomarker signature development, including supervised and unsupervised machine learning, feature selection and hypothesis testing approaches. In contrast to previous guidelines discussing detailed aspects of quality control, statistics or study reporting, we give a broader overview of the typical challenges and sort the quick tips to address them chronologically by the study phase (starting with study design, then covering consecutive phases of biomarker signature discovery and validation, see also the overview in Fig 1). While these tips are not comprehensive, they are chosen to cover what we consider as the most frequent, significant, and practically relevant issues and risks in biomarker development. By pointing the reader to further relevant literature on the covered aspects of biomarker discovery and validation, we hope to provide an initial guideline and entry point into the more detailed technical and application-specific aspects of this field.

## Tip 1: Choose a suitable study design

A first step in the preparation of biomarker signature discovery studies is to define the scientific objective and scope clearly and in detail. Common pitfalls to avoid include imprecise goals such as vague primary and secondary biomedical outcomes to investigate or a loosely defined study scope in terms of subject inclusion and exclusion criteria. This can lead to an inappropriate feasibility and risk assessment, to misunderstandings between the collaborators, and ultimately to a delayed or unsuccessful implementation. The collaborators should therefore agree on, and precisely define, the key study design aspects well in advance, and jointly assess the feasibility and suitability of the planned design in relation to the study goals. Apart from the definition of the specific scope, objectives, and milestones, this also includes the choice of relevant experimental conditions to study (diseases/subtypes/treatments) or prior data to include (e.g., existing clinical and health record data), the selection of a suitable tissue pool/cell type(s) and measurement platform, the biological sampling design (i.e., how the samples will be collected, if not already available), the blocking design [2], and the measurement design (i.e., the arrangement of samples in the measurement instrument and across different measurement batches [3]). Moreover, to ensure that the study is adequately powered and that biospecimen resources are used efficiently, dedicated sample size determination methods [4] and sample selection and matching methods (e.g., for confounder matching between cases and controls) [5] should be applied.

Studies that aim to assess the effects of interventions should include potential confounders as covariates. However, covariates that are common effects of treatment and outcome should not be included in the analysis because they would lead to selection and collider bias [6,7]; likewise, it is not recommended to indiscriminately include pretreatment covariates as they can induce bias amplification [6–8]. In contrast, studies that are purely predictive, without an interest in causation, do not have to be concerned about confounders, and the criteria of covariate inclusion purely depend on increasing predictive performance (see also Tips 4 to 8). Additionally, a specific and common concern with covariates in these types of studies is understanding the relative contribution of different types of variables, in particular clinical versus omics variables, which we address in Tip 3.

As part of the study design, early planning is required to ensure that legal and ethical requirements of data collection will be met throughout the study. For maintaining data security and privacy, data management and access strategies should be defined during this initial planning phase, e.g., by following specific frameworks and guidelines for this purpose [9,10]. Finally, a comprehensive and clear documentation of the study design is essential for effective project monitoring. For this purpose, we recommend following standard reporting guidelines, including visual illustrations of the study design or patient flow through the study, such as CONSORT [11] or STARD [12,13].

## Tip 2: Ensure data quality, curation, and standardization

Many biomedical datasets derived from non-targeted molecular profiling or high-throughput imaging approaches are affected by multiple sources of noise and bias, and clinical datasets are often not harmonized across different patient cohorts. In general, one can distinguish between technical noise and biological variance. Current data analytical methods have only a limited ability to discriminate between them. Therefore, quality control and filtering analyses, data curation, annotation, and standardization are important initial steps in biomedical data processing pipelines. Relevant quality controls typically include statistical outlier checks and computing data type-specific quality metrics, as implemented in established software packages, e.g., the *fastQC/FQC* package for next-generation sequencing (NGS) data [14], *arrayQualityMetrics* for microarray data [15], *pseudoQC*, *MeTaQuaC*, and *Normalyzer* for proteomics and metabolomics data [16–18]. Further dedicated quality assurance methods have been developed for cellular and neuroimaging data [19,20], clinical data [21,22], and digital biomarkers [23]. All quality checks should be applied both before and after preprocessing of the raw data to ensure that all quality issues have been resolved and no artificial patterns were introduced by inadequate preprocessing methods.

Apart from the initial processing and filtering, the curation of clinical data also involves dedicated checks and data transformations, e.g., ensuring that the values fall within acceptable ranges (e.g., checking maximum and minimum age and body mass index values), resolving inconsistencies (e.g., different units or value encodings), and transforming the data to standard formats (e.g., OMOP [24], CDISC [25], ICD10/11 [26], SNOMED CT [27]). Beyond these curation steps, a minimum set of required complementary annotations should be made available for subsequent data analyses and dissemination. Standard formats for providing annotations for the most common experimental and clinical data types have already been established, e.g., the MIAME [28] and MINSEQE [28,29] guidelines for microarray and NGS experiments and corresponding standards for metabolomics and proteomics data (e.g., MIAPE [30] and MSI [31]). These standards should be adopted already in the early data processing stages.

Finally, as part of the data curation and standardization, it is recommendable to compare and evaluate multiple options to define primary and secondary study endpoints and other key input and outcome variables (e.g., comparing different definitions of tumor grades or disease stages or different disease ontologies [32]). Considering multiple definitions of the same disease outcomes can help to address lack of clarity or loss of information associated with the use of only a single outcome definition.

## Tip 3: Integrate different data types effectively and assess the value of clinical versus omics data

Studies that have access to multiple datasets or use variables of qualitatively different kinds (e.g., clinical and omics) need to integrate these data effectively. In the machine learning literature, traditionally 3 different strategies for multimodal data integration have been suggested, namely early, intermediate, and late integration [33,34]. Early integration methods focus on extraction of common features from several data modalities. A typical example is canonical correlation analysis (CCA) and sparse variants of CCA [35,36]. In a second step, conventional machine learning methods can then be applied based on the extracted common feature space.

Late integration algorithms first learn separate models for each data modality and then combine predictions made by these models, for example, with the help of a meta-model trained on the outputs of data source specific sub-models. The latter strategy is called stacked generalization, stacking, or super learning [37–39].

Intermediate integration algorithms are the youngest branch of data fusion approaches. The idea is to join data sources while building the predictive model. A classic example of this strategy is support vector machine (SVM) learning with linear combinations of multiple kernel functions [34]. More recently, multimodal neural network architectures have been devised for this purpose [40].

A related problem to data integration is the selection of the most useful data type(s), when multiple available datasets contain redundant information, but have different informative value. A common example for this in biomedicine is assessing the clinical utility of omics data, or any other type of high-dimensional experimental measurement data, when we already have data from traditional clinical markers. The key question here is whether predictors built from omics data provide an added value for decision-making. Addressing this question requires comparative evaluations in addition to an integrative analysis and using the traditional clinical data as the baseline [41–44].

For more detailed guidelines and relevant method comparisons, we refer the reader to a broader overview of machine learning methods for omics data integration [45], representative case studies on combining omics and clinical data [46], and generic multi-omics integration approaches [47,48].

## Tip 4: Choose adequate preprocessing and filtering approaches

Raw biomedical data is often influenced by a variety of preanalytical factors, resulting in systematic biases and a shifting and scaling of the measured signals. Many artifacts and normalization issues are data type specific and need to be addressed using dedicated preprocessing and filtering methods. Tailored software solutions have been made available to preprocess clinical data [21], NGS data [49], microarray data [50], different types of metabolomics and proteomics data [18], and cellular and brain imaging data [51–54]. Although no generic rules and methods exist for all data types, the following considerations apply to most datasets. For attributes with a large proportion of missing values (e.g., more than 30% of values missing), researchers may want to consider a complete removal. For features with smaller numbers of missing values, imputation methods or machine learning algorithms that tolerate a limited occurrence of missing values may be applied, depending on the type of missingness [55]. To filter out uninformative attributes, the removal of features with zero or small variance is also recommended, and further alternative filtering methods using the sum of absolute covariances [55,56] or tests of the unimodality or multimodality of the data distribution have been proposed [57]. After filtering, additional standardization, transformation, or scaling steps may also be warranted. For example, standardization can help to make clinical features on different scales more comparable, and, for linear models, assumptions about the linearity, distribution, and constant variance of the response are often better met after using transformations such as Box-Cox [58,59]. Moreover, functional omics data often displays a dependence of the feature signal variance on the average signal intensity, which can be addressed by a variance stabilizing transformation [60–62]. Finally, the successful application of data filtering and preprocessing should be checked and evaluated, e.g., by repeating initial quality control analyses (see Tip 2) and assessing global shape and distribution characteristics of the processed data using low-dimensional visualizations (e.g., principal coordinate analysis [63], non-metric multidimensional scaling [64], t-SNE [65], and UMAP [66]) and dedicated software tools for omics visualization [67].

## Tip 5: Compare and select relevant modeling methods

After data preprocessing, appropriate statistical and machine learning methods need to be chosen for the analysis. Model selection strongly depends on the analysis goals, e.g., whether a probabilistic model of the data or a prediction of a categorical outcome is needed, and whether the study focus is on model interpretability or model performance. To preselect suitable algorithms for comparative evaluation, the number of input and output features, the number of available samples, and the type of features (categorical, numerical, ordinal) need to be considered [57,68]. The selection of the modeling procedure can also be informed by low-dimensional data visualizations and distribution plots [69–71]. However, low-dimensional intuitions of patterns in high-dimensional data can also be misleading, if the sample distances in the original feature space are not well preserved and partly reflect idiosyncrasies of the visualization method [72]. To facilitate model selection for the non-expert, automated machine learning (AutoML) approaches have been proposed, which use combinatorial search algorithms and heuristics to replace manual tasks in model selection [73]. But not all models are suitable for all types of data. For example, training a deep neural network with high-dimensional data of a few hundred patient samples is likely to result in a highly overfitted model. Hence, it is necessary to carefully choose the right types of models a priori and not purely rely on brute force compute power. To facilitate the choice for the reader, an overview of commonly used unsupervised and supervised machine learning algorithms, including popular implementations in the programming languages R and Python, references to methodology descriptions, and best practice example applications is provided in Tables A and B in S1 Text, respectively.

Once suitable modeling procedures have been chosen, comparing multiple representative approaches is recommended. This can be achieved by applying cross-validation or bootstrapping methods, followed by comparing different performance metrics using statistical tests [74,75] (see also Tip 6). However, overfitting should be avoided, e.g., by using nested cross-validation, and the significance scores for performance statistics should be adjusted for multiple hypothesis testing [75]. Apart from *p*-value significance scores, confidence intervals and similar measures of uncertainty should be assessed [76–79], taking into account the limitations of individual uncertainty measures [80]. Finally, in addition to assessing individual machine learning algorithms, the integration of modeling approaches using ensemble learning (for both supervised and unsupervised problems) or consensus clustering (for unsupervised problems) may be explored to combine the benefits of different modeling methods [81,82].

While extensive model evaluations and comparisons are generally beneficial, the success and feasibility of the model selection scheme will also depend on realistic time planning and consideration of the run-time requirements for the preselected algorithms [83]. At the end of a comparative model evaluation, several algorithms may display a very similar prediction performance. Hence, secondary selection criteria, such as interpretability or stability of feature selection should be considered. In summary, researchers should carefully plan all model selection steps and choose suitable and objective evaluation criteria before running computationally expensive analyses.

## Tip 6: Optimize model parameters and feature selection without overfitting

Biomedical datasets often have many more features than samples (the “p >> n” problem). This increases the risk for creating overfitted models, because data points are sparsely distributed in a very high dimensional space, resulting in statistically unstable models. Two popular approaches to prevent overfitting are ridge and lasso regularization [84,85], which shrink the squared, or respectively, absolute model coefficients towards zero. Alternatively, combining ridge and lasso regularization, the elastic net [85,86] can handle correlated variables more effectively than the lasso [85,87]. By optimizing the regularization parameter, which determines the extent to which estimated model coefficients are shrunk towards zero, we can prevent overfitting (too little shrinkage) and underfitting (too much shrinkage). The most common way of optimizing this and other hyperparameters is to perform a grid search with cross-validation, but there are more efficient alternatives [88,89], as well as Bayesian procedures, in which the prior performs the role of the penalty [90–92].

A common mistake in model optimization is to not only perform unsupervised but also supervised feature selection outside cross-validation. For example, removing features because of their low variance or their high correlation with other input features is a suitable global filtering method, but removing features from both training and test set data because of their low correlation with the target variable is an error [84]. Supervised attribute selection must take place inside cross-validation to avoid information leakage and overoptimistic estimates of predictive performance resulting from selection bias [93,94]. This also applies if the aim is to compare different approaches (e.g., data pre-preprocessing, feature transformation) before selecting the most predictive one. Moreover, if cross-validation is applied for both hyperparameter optimization and performance estimation (see Tip 7), a nested cross-validation scheme is required, i.e., while an outer cross-validation loop is used for performance estimation, an inner cross-validation loop is used for hyperparameter optimization. An alternative to selecting single hyperparameters by cross-validation is to combine multiple hyperparameters by stacked generalization [37,95,96]. Furthermore, predictive models avoiding explicit hyperparameter optimization may be chosen, e.g., random forests [97–99].

Finally, for many biomedical applications, natural structures among features or complementary information on the features can be exploited as an additional information source for model building. For example, among causally related features, we might want to prioritize the selection of upstream over downstream features in a known causal graph [100] to account for pairs or groups of functionally related features [101,102] or to transfer information from previous studies (i.e., prior weights or prior effects) into the learning procedure. These approaches to integrate prior knowledge into the learning phase have the potential to render models more predictive and more interpretable.

## Tip 7: Assess model performance in an unbiased and robust fashion

Once the data have been prepared and modeling approaches selected, a metric has to be chosen to assess model performance. The performance metric selection is problem specific, and it is often recommended to consider multiple metrics to distinguish between different error types (e.g., type 1 versus type 2 error) and consider different penalties for outliers (e.g., quadratic versus non-quadratic loss functions). This is particularly important for imbalanced study groups [103], often observed in biomedical projects (e.g., identifying approximately 0.3% breast cancer patients in a population-wide mammography screening). Researchers may consider using balanced accuracy measures or ensure balancing during model training by applying over/undersampling or data augmentation methods (test set samples should however always remain independent from the training set and synthetic redundancy introduced by oversampling should be avoided) [104–106]. Moreover, a prior sample size calculation and clearly defined study goals can help to ensure that enough samples for each study group are available for both modeling and performance assessment. In general, researchers should ensure that machine learning models are well calibrated, i.e., the distribution of predicted probabilities is close to the true probabilities of class membership. The most common calibration techniques and calibration measures for this purpose have been reviewed previously [107].

Common performance measure choices include the balanced accuracy, the F1 score, Matthew’s correlation coefficient, sensitivity/specificity for supervised binary classification, the mean squared error or absolute error and (adjusted) R^2^ for regression tasks [59,84,92], and internal validity indices, such as the average Silhouette width or Calinski–Harabasz index for unsupervised clustering [108,109]. However, the choice of the performance metric does not only depend on the outcome variable type but also the specific analysis goals and applications (see [110] for an empirical study of different performance metrics). Moreover, for classifiers that provide predicted probabilities for group membership rather than pure categorical outcome predictions, dedicated performance measures are available to avoid the subjective choice of threshold values for outcome categorization (a problem that affects accuracy, sensitivity, and specificity measures [111,112]). These include Brier’s score, the concordance index, the area under the receiver operating characteristic curve (AUC), the precision-recall curve (PR AUC), and the kappa curve (AUK), which can also be applied to survival data [111–116]. Depending on the clinical scenario, the uniform weighting of type 1 and type 2 errors in classical performance measures may sometimes provide counterintuitive classifier rankings, and the use of decision-analytic tools, which take into account the costs of different error types, should be considered [112,117].

When estimating a model’s generalization performance from observational data, the variability in biomedical datasets is often high, due to both technical and biological sources of variation. To address this challenge, bootstrapping methods, such as .632+ bootstrap, can be used to obtain more robust performance estimates [118]. Another well-accepted approach is repeated or iterated *k*-fold cross-validation, which often gives less biased estimates of the true generalization performance [119]. When selecting the parameter *k*, the user should be aware of the balance between bias (low *k*) and variability (high *k*, e.g., for leave-one-out cross-validation) [118,120]. Bolstered error estimation is a further robust alternative approach dedicated specifically to datasets with small sample size [121,122]. Finally, it is important to remember that high estimated performance on a single test dataset does not equate to generalizability on other datasets and to clinical or biomedical relevance [123] (see also Tip 8). More detailed practical guidance on the use of relevant algorithms and software tools for model performance assessment, including best practice examples, is provided in [85,92,124–126].

## Tip 8: Improve and validate the generalization capability of the model

Depending on the goals of a biomarker study (e.g., whether the study involves a clinical validation or only preclinical biomarker research) and the study type (e.g., whether the study is prospective or retrospective), different options are available to improve and evaluate an initial biomarker signature obtained from a discovery cohort. Clinical biomarker studies require that the final model is locked and recorded before testing on an independent validation cohort. The subjects in the validation cohort have to be representative of the intended patient population and fulfill the same inclusion and exclusion criteria as the discovery cohort [127,128]. Depending on whether the discovery and validation cohorts cover distinct geographic regions, environments, and ethnic backgrounds, the generalization capability of the final model may be restricted significantly by the population coverage and diversity of the included cohorts.

Studies focusing on early preclinical stages of biomarker discovery have more flexibility in collecting additional data to optimize and confirm the generalization capability of an initial machine learning model. Apart from straightforward optimization strategies, such as increasing the size of the discovery cohort and thereby the size of the training dataset for modeling, a wide range of external data sources can be exploited to further improve a model. For example, integrative meta-analyses of in-house data and relevant public or collaborator-derived clinical and omics data can be applied to improve the feature selection for a model [129], or prior knowledge from cellular pathway databases and the biomedical literature can be used to filter predictive molecular biomarkers depending on their involvement in disease-associated pathways [130] or to derive more robust pathway- or network-based predictive features [131]. Furthermore, cellular or animal models for the disease condition of interest can provide additional data for biomarker validation, which is often freely available in public data repositories. Functional validation studies involving the modulation of candidate biomarker molecules or pathways via knockdown and overexpression experiments in a disease model may provide information on causal associations with measurable disease phenotypes [132]. While all these information sources provide effective means for the initial confirmation and filtering of candidate markers, after having optimized a biomarker signature and locked down the final machine learning model, the final clinical evaluation will always require an adequately powered external validation on a distinct, representative patient cohort.

## Tip 9: Ascertain that the model meets the required level of interpretability and explainability

Depending on the goals of a biomedical prediction or stratification project, the success of applied machine learning methods might not only depend on the predictive performance of generated models but also their interpretability, biological plausibility, and insightfulness. When interpretability and explainability are relevant objectives and criteria for the study success, researchers should consider so-called “white-box” learning algorithms, i.e., modeling approaches that link input features to the outcome variable of interest in a more transparent and easier to understand fashion than the more complex, but often also more accurate, “black-box” modeling methods.

For settings requiring a high level of model interpretability, a wide variety of machine learning approaches is available to find a suitable compromise between model generalization capability and explainability. Common examples for learning approaches favoring interpretability are linear modeling methods [92] and rule-based machine learning methods, such as classification and regression trees [133,134], combinatorial rule learning approaches [135,136], and probabilistic and fuzzy rule learning methods [137,138]. While linear modeling approaches enable a relevance scoring and ranking of features by their absolute weights in a model, rule-based learning approaches can provide additional information on feature associations by computing statistics on their co-occurrence in decision rule sets [139]. Apart from these generic learning methods, more recently, domain-specific interpretable prediction and clustering approaches, which exploit prior biological knowledge from cellular pathways and molecular networks [140–142], have gained interest. In addition, there is a quickly growing literature on Explainable AI (XAI) techniques to interpret also very complex black-box models, such as neural networks. Examples include Shapley Additive Explanations [143], LIME [144], Explainable Boosting Machines [145], and symbolic meta-modeling [146]. A systematic review of those and further methods can be found in [147,148].

In summary, white-box modeling methods are not required for all applications, but being able to understand a stratification or prediction model derived from biomedical data and assess its biological plausibility is often beneficial, and particularly important in clinical decision support applications. In these settings, the transparency, credibility, and trustworthiness of machine learning models is equally important as the evidence for predictive power [149].

## Tip 10: Translate biomarker discoveries to in vitro diagnostics or diagnostic medical devices

Most biomarker signature discoveries are obtained using non-targeted, high-throughput measurement approaches, which cover large numbers of candidate biomarkers, but lack sensitivity and are not certified for diagnostic applications. If the long-term study goal is to develop biomarker findings into a clinically validated diagnostic test, then it is typically not only necessary to validate the biomarker signature on an external cohort but also to translate the original high-throughput measurement approach to a more targeted and sensitive measurement technology, which fulfills the requirements for clinical biomarker applications in terms of technical reliability and robustness.

Typical examples for this transition from non-targeted methodologies (e.g., omics profiling of patient biospecimens) to a targeted approach are the replacement of high-throughput transcriptomics profiling by targeted qRT-PCR or digital PCR measurements, or the replacement of mass spectrometry (MS)-based proteomics by targeted immunoassays, after developing and producing specific antibodies targeting the omics-derived peptide or protein fragment biomarkers. While the original discovery analyses are conducted on measurements for several thousands of biomarkers (e.g., 50k genetic transcripts), the targeted analyses focus only on small numbers of candidate biomarkers (e.g., 10 to 20 transcripts), selected using machine learning and cross-validation analyses of the original discovery data. The transition from non-targeted to targeted approaches normally does not only just require a new validation of the targeted version of the biomarker signatures but also adjustments of model parameters. If sufficient training data is available for the targeted method according to a sample size calculation, this model adjustment can be obtained by simply refitting the model on the new data. However, to guide the model building process and exploit the prior data from non-targeted analyses, it may additionally be worthwhile to consider applying transfer learning approaches. Transfer learning techniques use information from pre-trained machine learning models (e.g., information on the feature relevance or feature effects with respect to a clinical outcome of interest) to apply it to a new but similar data analysis task, in order to exploit the prior information to build more robust and accurate models (see [150] for a review of methodologies).

After a biomarker model has been refitted successfully to targeted measurement data, there are 2 main possible pathways for translating the model into a clinical biomarker test: The development of an in vitro diagnostic (IVD) or a diagnostic medical device. IVDs are tests applied to human body fluid or tissue samples to assess an individual’s health status. In contrast to other medical devices, they do not involve any direct action on the patient. By contrast, diagnostic medical devices can come in direct contact with the patient and include active devices with different levels of associated risk (in different countries, medical devices are categorized into different regulatory classes, depending on the risk and the required regulatory control). In the EU, all medical devices must be CE marked before they can reach the market (“CE” stands for “conformité européenne” and indicates that a product has been assessed by the manufacturer and deemed to meet EU safety, health, and environmental protection requirements). Further details on regulatory pathways for machine learning-based IVD and diagnostic medical device development and a comparison of associated policies in Europe and the United States can be found in a dedicated article [151]. Finally, researchers should take into consideration relevant FDA guidelines, in particular the “Good Machine Learning Practice for Medical Device Development: Guiding Principles” [152], which highlights the different types of multidisciplinary expertise required throughout the total product life cycle of a medical device.

## Conclusions

Biomarker signature discovery and development involve complex interdisciplinary collaborations and several interdependent tasks and decisions, ranging from the initial choice of study design parameters to the approaches for data collection and preprocessing, and the strategies for model building and validation. Many of the challenges in these projects are study and problem specific and cannot be fully addressed by general guidelines and recommendations. However, a variety of common pitfalls, issues, and limitations are shared across the majority of biomarker discovery and validation studies, and dedicated strategies and methods to circumvent or alleviate these common problems are already available.

In this article, we have chronologically summarized some of the most frequent challenges that occur during the typical phases of biomarker projects and suggested methods and software tools that may help to avoid unsuitable study designs, prevent analysis and validation errors, and increase chances for success. Since the practical implementation for many of the covered topics would require more detailed explanations, we have directed the reader to relevant literature with more in-depth information for each tip. For an overview of related existing guidelines and data and methods standardization efforts, we also recommend to study the “Criteria for the use of omics-based predictors in clinical trials” by the US National Cancer Institute [153] with a focus on omics-derived biomarkers and the standard framework “Assessing Credibility of Computational Modeling through Verification and Validation: Application to Medical Devices” with a broad applicability beyond the specific framework focus on medical devices [154]. Furthermore, as a guidance on how to document and present biomarker results derived from machine learning approaches, we refer the reader to the TRIPOD Statement on “Transparent reporting of a multivariable prediction model for individual prognosis or diagnosis” [155] and the more generic “Standards for Reporting of Diagnostic Accuracy (STARD)” [12,13]. In practice, project managers should also ensure that the required multidisciplinary expertise for all project phases is well represented in the project consortium, and that measures for effective cross-disciplinary communication throughout the project are set in place.

As further steps in the future, community-driven standardization efforts, involving researchers, practitioners, and regulators in the field, are still needed to develop more comprehensive and detailed documentation and validation standards, minimum requirements, and study type-specific guidelines to further improve the quality of biomarker stratification and prediction projects.

## Supporting information

S1 Text Supporting Text S1 for the manuscript “Ten Quick Tips for Biomarker Discovery and Validation Analyses Using Machine Learning”. Table A in S1 Text. Unsupervised learning algorithms. Overview of widely used unsupervised machine learning algorithms, including implementations in the programming languages R and Python, references to methodology descriptions, and best practice example applications. **Table B in S1 Text. Supervised learning algorithms.** Overview of widely used supervised machine learning algorithms, including implementations in the programming languages R and Python, references to methodology descriptions, and best practice example applications.
(PDF)
