# Supplementary material for: Ten quick tips for biomarker discovery and validation analyses using machine learning
Source: PLoS Comput Biol. 2022 Aug 11;18(8):e1010357. doi: 10.1371/journal.pcbi.1010357 (PMC9371329; doi:10.1371/journal.pcbi.1010357)
Supplement: S1 Text — Table A in S1 Text. Unsupervised learning algorithms. Overview of widely used unsupervised machine learning algorithms, including implementations in the programming languages R and Python, references to methodology descriptions, and best practice example applications. Table B in S1 Text. Supervised learning algorithms. Overview of widely used supervised machine learning algorithms, including implementations in the programming languages R and Python, references to methodology descriptions, and best practice example applications. (PDF) [file pcbi.1010357.s001.pdf]

Supporting Text S1 for the manuscript "Ten Quick Tips for Biomarker Discovery and Validation Analyses Using Machine Learning"

Table A: Unsupervised learning algorithms

**Name**	**Methodology**	**Implementation** Packages and *functions*()	**Application**
Hierarchical clustering	- BIRCH (Zhang et al. 1997 [1]),<br>- Chameleon (Karypis et al. 1999 [2])<br>- Echidna (Mahmood et al., 2006 [3])<br>- PDDP (Boley, 1998 [4])<br>- DHCC (Boley et al, 1999 [5])	**R**:<br>● cluster<br>● stats::*hclust*()<br><br>**Python**:<br>● sklearn *AgglomerativeClustering*()	Eisen et al., 1998 [6]<br><br>Pagnuco et al., 2017 [7]
Partitioning-based clustering	- k-means (Hartigan & Wong, 1979 [8])<br>- k-medoids (Lucasius et al., 1993 [9])<br>- PAM (Kaufman & Rousseeuw, 1990 [10])<br>- CLARA (Kaufman & Rousseeuw, 2005 [11])<br>- CLARANS (Ng & Han, 2002 [12])	**R**:<br>● cluster<br>● stats::*kmeans*()<br><br>**Python**:<br>● sklearn *KMeans()*<br>● sklearn_extra *KMedoids()*<br>● pyclustering *clarans()*	Gasch & Eisen, 2002 [13]<br><br>Chappell et al., 2017 [14]
Model-based approaches	- EM (Dempster et al., 1977 [15])<br>- SOM (Kohonen, 1982 [16])<br>- COBWEB (Fisher, 1987 [17])	**R**:<br>● EMcluster<br>● mixtools<br>● kohonen<br><br>**Python**:<br>● sklearn *mixture()*<br>● sklearn_som	Quang & Xie, 2014 [18]<br><br>Deng et al., 2020 [19]
Density-based	- DBSCAN (Ester et	**R**:	Kinable &

methods	al. 1996 [20])<br>- OPTICS (Ankerst et al, 1999 [21])	● dbscan<br><br>**Python**:<br>● sklearn<br>*dbscan(), OPTICS()*	Kostakis, 2011 [22]<br><br>Edla & Jana, 2012 [23]
Graph-based clustering	- spectral clustering (Chung, 1996 [24]; Luxburg, 2007 [25])	**R**:<br>● Spectrum<br><br>**Python**:<br>● sklearn<br>*SpectralClustering()*	Li et al., 2021 [26]

<u>Table B: Supervised learning algorithms</u>

**Name**	**Methodology**	**Implementation**<br>Packages and *modules / functions()*	**Application**
Regularised regression	- GLM (McCullagh & Nelder, 1983, [27])<br>- ridge (Hoerl & Kennard, 1970, [28])<br>- lasso (Tibshirani, 1996, [29])<br>- elastic net (Zou & Hastie, 2005, [30])	**R**:<br>● glmnet<br><br>**Python**:<br>● sklearn.*linear_model*	Segal et al., 2003 [31]<br>Irwin et al., 2020 [32]<br>Liu et al., 2019 [33]<br>Ogutu et al., 2012 [34]
Decision trees	- Breiman et al. (1984) [35], Quinlan (1986) [36]	**R**:<br>● rpart<br><br>**Python**:<br>● sklearn.*tree*	Gifu, 2021 [37]<br><br>Tayefi et al., 2017 [38]
K-nearest neighbours (kNN)	- Fix & Hodges 1951 [39]	**R**:<br>● class::*knn()*<br><br>**Python**:<br>● sklearn.*neighbors*	Parry et al., 2010 [40]<br><br>Widiawati et al., 2018 [41]
Support vector machines (SVM)	- Cortes & Vapnik 1995 [42]	**R**:<br>● e1071::*svm()*<br><br>**Python**:<br>● sklearn.*svm*	Rapaport et al., 2008 [43]<br><br>Kim, 2016 [44]

Artificial neural networks	- McCulloch & Pitts, 1943 [45]<br>- Hopfield, 1988 [46]	**R**:<br>• neuralnet<br><br>**Python**:<br>• sklearn.*neural_network*	Liang and Kelemen, 2005 [47]<br><br>Dubost et al., 2019 [48]<br><br>Xu et al., 2016 [49]
Ensemble learning	- stacking (Wolpert 1992, [50])<br>- bagging (Breiman 1996, [51])<br>- boosting (Schapire 1990, [52])	**R**:<br>• SuperLearner<br>• randomForest<br>• xgboost<br>• varSelRF<br><br>**Python**:<br>• sklearn.*ensemble*	Tang et al., 2017 [53]<br>Liu et al., 2018 [54]<br>Fraz et al., 2012 [55]<br>Ubels et al., 2020 [56]<br>Diaz-Uriarte et al., 2007 [57]<br>Díaz-Uriarte & de Andrés, 2006) [58]<br>Pouyan & Kostka, 2018 [59]
Naïve Bayes	- Duda & Hart, 1973 [60]<br>- Hand et al., 2001 [61]	**R**:<br>• e1071::*naiveBayes()*<br><br>**Python**:<br>• sklearn.*naive_bayes*	Geng et al., 2015 [62]<br><br>Wei et al., 2011 [63]<br><br>Sambo et al., 2012 [64]

References

1. Zhang T, Ramakrishnan R, Livny M. Data Min Knowl Discov. 1997;1: 141–182.

2. Karypis G, Han E-H, Kumar V. Chameleon: hierarchical clustering using dynamic modeling. Computer. 1999;32: 68–75.

3. Mahmood AN, Leckie C, Udaya P. Echidna: Efficient clustering of hierarchical data for network traffic analysis. NETWORKING 2006 Networking Technologies, Services, and Protocols; Performance of Computer and Communication Networks; Mobile and Wireless Communications Systems. Berlin, Heidelberg: Springer Berlin Heidelberg; 2006. pp. 1092–1098.

4. Boley D. Principal direction divisive partitioning. Data Min Knowl Discov. 1998;2: 325–

344.

5. Boley D, Gini M, Gross R, Han EH, Hastings K, Karypis G, et al. Partitioning-based clustering for Web document categorization. Decis Support Syst. 1999;27: 329–341.

6. Eisen MB, Spellman PT, Brown PO, Botstein D. Cluster analysis and display of genome-wide expression patterns. Proc Natl Acad Sci U S A. 1998;95: 14863–14868.

7. Pagnuco IA, Pastore JI, Abras G, Brun M, Ballarin VL. Analysis of genetic association using hierarchical clustering and cluster validation indices. Genomics. 2017;109: 438–445.

8. Hartigan JA, Wong MA. Algorithm AS 136: A K-means clustering algorithm. J R Stat Soc. 1979;28: 100–108.

9. Lucasius CB, Dane AD, Kateman G. On k-medoid clustering of large data sets with the aid of a genetic algorithm: background, feasiblity and comparison. Anal Chim Acta. 1993;282: 647–669.

10. Kaufman L, Rousseeuw PJ. Partitioning around medoids (program PAM). Finding Groups in Data. Hoboken, NJ, USA: John Wiley & Sons, Inc.; 2008. pp. 68–125.

11. Kaufman L, Rousseeuw PJ. Finding Groups in Data: An Introduction to Cluster Analysis. Wiley-Interscience; 2005.

12. Ng RT, Han J. CLARANS: a method for clustering objects for spatial data mining. IEEE Trans Knowl Data Eng. 2002;14: 1003–1016.

13. Gasch AP, Eisen MB. Exploring the conditional coregulation of yeast gene expression through fuzzy k-means clustering. Genome Biol. 2002;3: RESEARCH0059.

14. Chappell T, Geva S, Hogan J. K-means clustering of biological sequences. Proceedings of the 22nd Australasian Document Computing Symposium. New York, NY, USA: ACM; 2017. doi:10.1145/3166072.3166076

15. Dempster AP, Laird NM, Rubin DB. Maximum likelihood from incomplete data via the EM algorithm. J R Stat Soc. 1977;39: 1–22.

16. Kohonen T. Self-organized formation of topologically correct feature maps. Biol Cybern. 1982;43: 59–69.

17. Fisher DH. Knowledge acquisition via incremental conceptual clustering. Mach Learn. 1987;2: 139–172.

18. Quang D, Xie X. EXTREME: an online EM algorithm for motif discovery. Bioinformatics. 2014;30: 1667–1673.

19. Deng W, Mou T, Kalari KR, Niu N, Wang L, Pawitan Y, et al. Alternating EM algorithm for a bilinear model in isoform quantification from RNA-seq data. Bioinformatics. 2020;36: 805–812.

20. Ester, M. Kriegel, H.-P. Sander, J. Xu, X. A density-based algorithm for discovering clusters in large spatial databases with noise. In: Simoudis E., Han J., Fayyad U., editor. KDD'96: Proceedings of the Second International Conference on Knowledge Discovery and Data Mining. 1996. Available: https://dl.acm.org/doi/abs/10.5555/3001460.3001507

21. Ankerst M, Breunig MM, Kriegel H-P, Sander J. OPTICS. Proceedings of the 1999 ACM

SIGMOD international conference on Management of data - SIGMOD ’99. New York, New York, USA: ACM Press; 1999. doi:10.1145/304182.304187

22. Kinable J, Kostakis O. Malware classification based on call graph clustering. J Comput Virol. 2011;7: 233–245.

23. Damodar Reddy E, Prasanta K J. A prototype-based modified DBSCAN for gene clustering. Procedia Technol. 2012;6: 485–492.

24. Chung F. Spectral Graph Theory. CBMS Regional Conference Series in Mathematics. 1996. doi:10.1090/cbms/092

25. Luxburg U von. A tutorial on spectral clustering. Stat Comput. 2007;17: 395–416.

26. Li Y, Luo P, Lu Y, Wu F-X. Identifying cell types from single-cell data based on similarities and dissimilarities between cells. BMC Bioinformatics. 2021;22: 255.

27. McCullagh P, Nelder JA. Generalized Linear Models. Routledge; 2019.

28. Hoerl AE, Kennard RW. Ridge regression: Biased estimation for nonorthogonal problems. Technometrics. 1970;12: 55.

29. Tibshirani R. Regression shrinkage and selection via the lasso. J R Stat Soc. 1996;58: 267–288.

30. Zou H, Hastie T. Regularization and variable selection via the elastic net. J R Stat Soc Series B Stat Methodol. 2005;67: 301–320.

31. Segal MR, Dahlquist KD, Conklin BR. Regression approaches for microarray data analysis. J Comput Biol. 2003;10: 961–980.

32. Irwin DJ, Fedler J, Coffey CS, Caspell-Garcia C, Kang JH, Simuni T, et al. Evolution of Alzheimer’s disease cerebrospinal fluid biomarkers in early Parkinson's disease. Ann Neurol. 2020;88: 574–587.

33. Liu S, Lu M, Li H, Zuo Y. Prediction of gene expression patterns with generalized linear regression model. Front Genet. 2019;10: 120.

34. Ogutu JO, Schulz-Streeck T, Piepho H-P. Genomic selection using regularized linear regression models: ridge regression, lasso, elastic net and their extensions. BMC Proc. 2012;6 Suppl 2: S10.

35. Breiman L, Friedman JH, Olshen RA, Stone CJ. Classification and regression trees. Wadsworth & Brooks. Cole Statistics/Probability Series. 1984.

36. Quinlan JR. Induction of decision trees. Mach Learn. 1986;1: 81–106.

37. Gifu D. The use of decision trees for analysis of the epilepsy. Procedia Comput Sci. 2021;192: 2844–2853.

38. Tayefi M, Esmaeili H, Saberi Karimian M, Amirabadi Zadeh A, Ebrahimi M, Safarian M, et al. The application of a decision tree to establish the parameters associated with hypertension. Comput Methods Programs Biomed. 2017;139: 83–91.

39. Silverman BW, Jones MC. E. Fix and J.L. Hodges (1951): An important contribution to nonparametric discriminant analysis and density estimation: Commentary on Fix and Hodges (1951). Int Stat Rev. 1989;57: 233–238.

40. Parry RM, Jones W, Stokes TH, Phan JH, Moffitt RA, Fang H, et al. k-Nearest neighbor models for microarray gene expression analysis and clinical outcome prediction. Pharmacogenomics J. 2010;10: 292–309.

41. Widiawati IF, Nugrahapraja H, Fajriyah R. K-Nearest Neighbor (KNN) Analysis on Genes Expression Datasets of Maize Nested Association Mapping (NAM) Showed Confident Classification on Organ-specific Expression. 2018 1st International Conference on Bioinformatics, Biotechnology, and Biomedical Engineering - Bioinformatics and Biomedical Engineering. 2018. doi:10.1109/biomic.2018.8610577

42. Cortes C, Vapnik V. Support-vector networks. Mach Learn. 1995;20: 273–297.

43. Rapaport F, Barillot E, Vert J-P. Classification of arrayCGH data using fused SVM. Bioinformatics. 2008. pp. i375–i382. doi:10.1093/bioinformatics/btn188

44. Kim S. Weighted K-means support vector machine for cancer prediction. Springerplus. 2016;5: 1162.

45. McCulloch WS, Pitts W. A logical calculus of the ideas immanent in nervous activity. Bull Math Biophys. 1943;5: 115–133.

46. Hopfield JJ. Artificial neural networks. Circuits Syst Mag IEEE. 1988;4: 3–10.

47. Liang Y, Kelemen A. Temporal gene expression classification with regularised neural network. Int J Bioinform Res Appl. 2005;1: 399–413.

48. Dubost F, Adams H, Bortsova G, Ikram MA, Niessen W, Vernooij M, et al. 3D regression neural network for the quantification of enlarged perivascular spaces in brain MRI. Med Image Anal. 2019;51: 89–100.

49. Xu J, Xiang L, Liu Q, Gilmore H, Wu J, Tang J, et al. Stacked Sparse Autoencoder (SSAE) for Nuclei Detection on Breast Cancer Histopathology Images. IEEE Trans Med Imaging. 2016;35: 119–130.

50. Wolpert DH. Stacked generalization. Neural Netw. 1992;5: 241–259.

51. Breiman L. Bagging predictors. Mach Learn. 1996;24: 123–140.

52. Schapire RE. The strength of weak learnability. Mach Learn. 1990;5: 197–227.

53. Tang Y, Liu D, Wang Z, Wen T, Deng L. A boosting approach for prediction of protein-RNA binding residues. BMC Bioinformatics. 2017. doi:10.1186/s12859-017-1879-2

54. Liu X-J, Gong X-J, Yu H, Xu J-H. A model stacking framework for identifying DNA binding proteins by orchestrating multi-view features and classifiers. Genes. 2018;9: 394.

55. Fraz MM, Remagnino P, Hoppe A, Uyyanonvara B, Rudnicka AR, Owen CG, et al. An ensemble classification-based approach applied to retinal blood vessel segmentation. IEEE Trans Biomed Eng. 2012;59: 2538–2548.

56. Ubels J, Schaefers T, Punt C, Guchelaar H-J, de Ridder J. RAINFOREST: a random forest approach to predict treatment benefit in data from (failed) clinical drug trials. Bioinformatics. 2020;36: i601–i609.

57. Diaz-Uriarte R. GeneSrF and varSelRF: a web-based tool and R package for gene selection and classification using random forest. BMC Bioinformatics. 2007;8.

doi:10.1186/1471-2105-8-328

58. Diaz-Uriarte R, de Andrés SA. Gene selection and classification of microarray data using random forest. BMC Bioinformatics. 2006;7. doi:10.1186/1471-2105-7-3

59. Pouyan MB, Kostka D. Random forest based similarity learning for single cell RNA sequencing data. Bioinformatics. 2018;34: i79–i88.

60. Duda, R, Hart P. Pattern Classification and Scene Analysis. Wiley; 1973.

61. Hand DJ, Yu K. Idiot's Bayes: Not So Stupid after All? Int Stat Rev. 2001;69: 385–398.

62. Geng H, Lu T, Lin X, Liu Y, Yan F. Prediction of protein-protein interaction sites based on naive bayes classifier. Biochem Res Int. 2015;2015: 1–7.

63. Wei W, Visweswaran S, Cooper GF. The application of naive Bayes model averaging to predict Alzheimer's disease from genome-wide data. J Am Med Inform Assoc. 2011;18: 370–375.

64. Sambo F, Trifoglio E, Di Camillo B, Toffolo GM, Cobelli C. Bag of Naïve Bayes: biomarker selection and classification from genome-wide SNP data. BMC Bioinformatics. 2012;13 Suppl 14: S2.
